# Evaluation of the Molecular Landscape of Pediatric Thyroid Nodules and Use of a Multigene Genomic Classifier in Children

**DOI:** 10.1001/jamaoncol.2022.1655

**Published:** 2022-06-09

**Authors:** Jean-Nicolas Gallant, Sheau-Chiann Chen, Carlos A. Ortega, Sarah L. Rohde, Ryan H. Belcher, James L. Netterville, Naira Baregamian, Huiying Wang, Jiancong Liang, Fei Ye, Yuri E. Nikiforov, Marina N. Nikiforova, Vivian L. Weiss

**Affiliations:** 1 Department of Otolaryngology–Head and Neck Surgery, Vanderbilt University Medical Center, Nashville, Tennessee; 2 Department of Biostatistics, Vanderbilt University Medical Center, Nashville, Tennessee; 3 School of Medicine, Vanderbilt University, Nashville, Tennessee; 4 Division of Surgical Oncology and Endocrine Surgery, Department of Surgery, Vanderbilt University Medical Center, Nashville, Tennessee; 5 Department of Pathology, Microbiology, and Immunology, Vanderbilt University Medical Center, Nashville, Tennessee; 6 Department of Pathology, University of Pittsburgh Medical Center, Pittsburgh, Pennsylvania

## Abstract

**Importance:**

Definitive diagnosis of a thyroid nodule in a child is obtained through diagnostic surgery. This is problematic because pediatric thyroid surgery is associated with higher rates of complications. In adults, preoperative molecular testing improves the management of thyroid nodules, but this has not been validated in children.

**Objective:**

To determine whether the molecular landscape of pediatric thyroid nodules is amenable to detection by a multigene genomic classifier (GC) test (ThyroSeq v3; Sonic Healthcare USA).

**Design, Setting, and Participants:**

This was a retrospective consecutive case series and GC testing of fine-needle aspiration (FNA) and formalin-fixed paraffin-embedded (FFPE) tissues from sequential pediatric thyroidectomies performed between January 2003 and December 2019 at a single tertiary academic medical center. The study included 95 patients (median [range] age, 16.3 [4.8 to 21.1] years; 75 [79%] female) who underwent surgery for a thyroid nodule.

**Interventions:**

A total of 118 thyroid nodule samples (95 FFPE, 23 companion FNAs) yielded informative next-generation sequencing data and multigene GC.

**Main Outcomes and Measures:**

The primary outcome was the determination of the pediatric thyroid molecular landscape. The secondary outcome was the diagnostic accuracy of the GC test for pediatric thyroid nodules.

**Results:**

Of the 95 patients, 75 (79%) were female, and the median (IQR) age was 16.3 (14.0-17.3) years. Next-generation sequencing confirmed the unique molecular landscape of malignant pediatric thyroid nodules (compared with adults), which is dominated by gene fusions (most commonly *RET* and *NTRK*), rare *BRAF*/*RAS* alterations, and no *TP53* or *TERT* promoter pathogenic variants. Several poorly differentiated thyroid cancers harbored *DICER1* variants. Benign nodules appeared to be almost exclusively associated with *TSHR* and *DICER1* alterations. The test demonstrated a 96% sensitivity (95% CI, 87%-99%) and 78% specificity (95% CI, 64%-88%). The negative predictive value was 95% (95% CI, 88%-98%) and the positive predictive value was 83% (95% CI, 74–89%). The concordance of GC between 23 pairs of matched FFPE and FNA tissues was 96%.

**Conclusions and Relevance:**

The study results of this retrospective consecutive case series suggest that the molecular landscape of pediatric nodules is unique but remains amenable to molecular classification. The multigene GC test, with high sensitivity and reasonably high specificity, represents a potential addition to the diagnostic workup of children with thyroid nodules and may decrease the use of diagnostic surgery.

## Key Points

**Question:**

Are pediatric thyroid nodules amenable to cancer prediction by genomic classification?

**Findings:**

In this retrospective study of 95 pediatric patients with thyroid nodules, surgical samples underwent next-generation sequencing and genomic classification. Testing defined the unique molecular landscape of pediatric thyroid nodules (which, as opposed to adults, comprised more frequent gene fusions and *DICER1* variants) and identified a sensitivity of 96% and specificity of 78% regarding cancer detection.

**Meaning:**

The study results suggest that although the molecular landscape of pediatric thyroid nodules is different than in adults, it remains amenable to multigene genomic classification, which may help prevent potentially unnecessary diagnostic surgeries.

## Teaser

This study examines whether the molecular landscape of pediatric thyroid nodules is amenable to detection by a multigene genomic classifier test.

## Introduction

The incidence of pediatric thyroid cancer is increasing, and it is now the second-most common adolescent malignant neoplasm.^1^ Thyroid nodules are less prevalent in children than in adults (approximately 2% vs 30%); however, they carry a greater risk of malignancy (approximately 25% vs 5%).^2,3^ Fine-needle aspiration (FNA) cytology is the most common diagnostic test for thyroid nodules, but results are often indeterminate, with subsequent malignancy rates of 20% to 50%.^3^ Because of the substantial probability of cancer, current recommendations encourage diagnostic surgery following indeterminate FNAs in children.^4^ Thyroidectomy, which has higher rates of complications in children than in adults, allows for histologic assessment, which is the reference standard for diagnosing a thyroid nodule.^4^

In adults with indeterminate FNAs, recommendations are for patients to undergo a second FNA or molecular testing instead of proceeding with surgery.^5^ Smaller-scale studies have examined the genetics of thyroid tumors in children, but comprehensive next-generation sequencing (NGS) studies are lacking.^6,7^ This knowledge gap prevents the use of molecular diagnostics in children with thyroid nodules.^4^ In this large retrospective study, we sought to assess the performance of a DNA/RNA NGS genomic classification (GC) test (ThyroSeq v3; Sonic Healthcare USA) in pediatric thyroid nodules.^8^ The goals were to (1) elucidate the molecular landscape of pediatric thyroid nodules and (2) determine whether they were amenable to malignant neoplasm detection by GC.

## Methods

### Study Population

Consecutive patients 21 years or younger who underwent thyroid surgery at Vanderbilt University Medical Center (VUMC) between January 2003 and December 2019 were included if they had available specimens for analysis.

### Study Design

This was a retrospective cross-sectional study in which VUMC surgical formalin-fixed paraffin-embedded (FFPE) tissues and companion FNAs underwent GC testing at the University of Pittsburgh Medical Center. Before sequencing, all cases were rereviewed by 2 pediatric thyroid pathologists (H.W. and J.L.) and 2 cytopathologists (V.W. and H.W.). Equivocal cases were reviewed by a third pathologist with thyroid expertise. The VUMC institutional review board approval included a waiver of informed consent owing to the retrospective and masked nature of this analysis. This study adheres to the Strengthening the Reporting of Observational Studies in Epidemiology (STROBE) and Standards for Reporting of Diagnostic Accuracy (STARD) reporting guidelines.^9,10^

All statistical analyses were conducted with R, version 4.1.1 (R Foundation), and statistical significance was assessed at a 2-sided 5% level. Race and ethnicity, self-reported in clinic intake forms, were gathered from the medical record. Additional information about variables, molecular testing, study outcomes, and statistical analyses can be found in the eMethods in the Supplement.

## Results

### Patients

Nodules from 95 patients, 75 (79%) of whom were female (median age, 16.3; range, 4.8–21.1 years; Table 1), successfully underwent NGS. Fifty nodules (53%) were malignant according to final pathology results (eTable 1 in the Supplement). Patients with benign or malignant pathology results did not differ significantly regarding preoperative parameters except for body mass index *z* score, thyroid stimulating hormone (TSH), and Bethesda category.^11,12^

**Table 1.** Patient Characteristics

Characteristic	No. (%)			*P* value
	All patients	Nodules^a^		
		Benign	Malignant	
No.	95	45	50	
Age at surgery, median (IQR), y	16.3 (14.0 to 17.3)	16.4 (14.4 to 17.5)	16.1 (13.6 to 17.1)	.12^b^
Sex				
Female	75 (79)	36 (80)	39 (78)	.81^c^
Male	20 (21)	9 (20)	11 (22)	
Race				
White	83 (87)	40 (89)	43 (86)	.67^c^
Other^d^	12 (13)	5 (11)	7 (14)	
Dominant nodule volume, median (IQR), cm^3^	4.3 (1.5 to 9.9)	4.7 (2.8 to 8.8)	3.8 (1.2 to 11.2)	.62^b^
Personal history of thyroid disease				
Yes	30 (32)	12 (27)	18 (36)	.33^c^
No	65 (68)	33 (73)	32 (64)	
Preoperative TSH level, median (IQR)	1.6 (0.7 to 2.8)	0.9 (0.3 to 2.0)	2.1 (1.0 to 3.3)	<.01^b^
Bethesda system for reporting thyroid cytopathology category				
I	0	0	0	<.01^e^
II	12 (27)	8 (53)	4 (14)	
III	7 (16)	4 (27)	3 (10)	
IV	4 (9)	3 (20)	1 (3)	
V	4 (9)	0	4 (14)	
VI	17 (39)	0	17 (59)	
BMI *z* score, median (IQR)	0.8 (−0.1 to 1.9)	0.6 (−0.4 to 1.5)	1.2 (0.1 to 2.1)	.04^b^

Abbreviations: BMI, body mass index (calculated as weight in kilograms divided by height in meters squared); TSH, thyroid-stimulating hormone.

^a^ Benign nodules included follicular adenoma and multinodular goiter; malignant nodules included papillary thyroid carcinoma (and variants), follicular thyroid carcinoma, poorly differentiated carcinoma, and noninvasive follicular thyroid neoplasm with papillary-like nuclear features.

^b^ Wilcoxon rank sum test.

^c^ Pearson χ^2^ test.

^d^ Other race or ethnicity includes Arabic, Black, East Asian, Hispanic, and Latino.

^e^ Fisher exact test.

### Molecular Landscape of Pediatric Thyroid Nodules

Comprehensive NGS enabled us to define the molecular landscape of pediatric thyroid nodules (eFigure 1 and eTable 2 in the Supplement). As opposed to adult thyroid cancer, we identified few *BRAF*/*RAS* pathogenic variants and many gene fusions in malignant pediatric nodules: 11 (22%) malignant nodules harbored *BRAF*/*RAS* pathogenic variants while 29 (58%) harbored a gene fusion.^13^ Fusions largely (26 of 29 [90%]) involved *RET* and *NTRK1/3* but were also found in *ALK*, *BRAF,* and *PPARG* (eFigure 2 in the Supplement). No alterations associated with high-risk adult thyroid cancer (eg, *TP53*) were identified.

Gene fusions were almost exclusively (28 of 29 [97%]) found in papillary carcinomas, with 1 poorly differentiated carcinoma harboring a *PAX8*-*PPARG* fusion. Patients harboring malignant nodules with fusions were significantly younger (median [IQR] age, 14.4 [11.2-16.5] years) than those with pathogenic variants (median [IQR] age, 16.8 [15.9–17.6] years; eTable 3 in the Supplement). Nodules with gene fusions also portended a higher tumor stage, more extensive lymph node disease, and aggressive pathologic features, such as lymphovascular invasion. Despite these differences, nodules with fusions and those with pathogenic variants had similar survival (eFigure 3 in the Supplement).

Benign nodules were mainly associated with functional *TSHR* variants (19 [42%]) as evidenced by increased nodular sodium-iodide symporter expression and lower preoperative serum TSH levels (eFigure 4 in the Supplement). Hotspot *DICER1* variants were identified in benign (6 [13%]) and malignant (4 [8%]) nodules, including follicular and poorly differentiated carcinomas (eFigure 5 in the Supplement).

### Test Performance

Ultimately, 127 samples (82%) from 95 patients yielded informative molecular data (eFigure 6 in the Supplement). Final test characteristics included a 96% sensitivity (95% CI, 87%-99%) and 78% specificity (95% CI, 64%-88%). The negative predictive value was 95% (95% CI, 88%-98%) and the positive predictive value was 83%; (95% CI, 74%-89%) (Table 2). The GC correctly identified 4 malignant nodules (8%) that were benign on cytology (eFigure 7 in the Supplement). The ability of the GC test to predict a malignant neoplasm was better than any combination of tested clinical factors (eTable 4 in the Supplement) and effective across various pathologies (eFigure 8 in the Supplement). Importantly, the 23 pairs of matching FNA/FFPE samples were highly concordant, and FNA GC sensitivity was 94% (95% CI, 73%-100%; eTable 5 in the Supplement).

**Table 2.** Performance of the Genomic Classifier Test in Pediatric Thyroid Nodules

Characteristic	Estimation, % (95% CI)
Sensitivity	96.0 (86.5-98.9)
Specificity	77.8 (63.7-87.5
PPV	82.8 (73.9-89.0)
NPV	94.6 (88.1-97.6)
AUC	95.9 (90.9-100.0)

Abbreviations: AUC, area under the curve; NPV, negative predictive value; PPV, positive predictive value.

Of the 37 test-negative samples, 2 (4%) were false negative (malignant on histopathology); both were low-risk differentiated cancers (eTable 6 in the Supplement). Among the 58 test-positive samples, 10 (22%) were benign (false positive) on final pathology; these nodules harbored an *NRAS* or *DICER1* pathogenic variant, gene fusion, or high copy number alteration (CNA) (eTable 7 in the Supplement).

## Discussion

This study reports the molecular landscape of pediatric thyroid nodules at its highest resolution to date. The study results suggest that gene fusions represent a dominant mechanism of pediatric thyroid cancer and that pathogenic variants (including *BRAF*/*RAS* and high-risk *TP53*/*TERT*) are rare in this age group.^6,7^ *DICER1* variants were identified in malignant (8%) and benign (13%) nodules. The precise role of *DICER1* in thyroid oncogenesis is not fully understood. We hypothesize that additional hits, such as CNAs, are required for transformation of these nodules.^14^

Although molecular testing is used to treat adult patients with thyroid nodules, to our knowledge, its performance and clinical use in children has not been determined.^5,8,11^ This study used a large cohort of pediatric thyroid nodules and found that the multigene GC test had high sensitivity (96%) and good specificity (78%) for discriminating benign from malignant nodules. The test yielded a negative predictive value of 95% and a residual cancer risk of 4% in test-negative nodules, which is similar to the cancer risk in benign FNAs.^11^ All false-negative cases were low-risk cancers by multiple criteria. The GC correctly identified several cancers that would have been missed by FNA.

This study’s findings have several implications. First, the comprehensive testing methods of the GC were able to detect the unique alterations characteristic of pediatric thyroid tumors, including gene fusions and *DICER1* alterations. While targets may be refined, as it stands, the GC offers a potential addition to the workup of children with thyroid nodules, which may allow practitioners to safely decrease diagnostic surgeries. Second, robust detection of targetable fusions (*NTRK*, *RET*) could help with targeted therapy in the case of poor outcomes (eg, progression following treatment with radioactive iodine) or in the neoadjuvant setting.^15^ Finally, the genomic information in this study could help expand understanding of pediatric thyroid (cancer) biology.

### Limitations

First, there could be a sampling bias in this retrospective study, which may have affected the proposed test characteristics. Second, this study was performed at a high-volume center with established thyroid expertise; thus, the results may not be generalizable to different settings. Additionally, the small number of patients of racial or ethnic minority groups included in this study limited generalizability to these underserved groups.

## Conclusions

The results of this retrospective case series suggest that the performance of GC for pediatric thyroid nodules may prevent diagnostic surgeries in a pediatric patient population. Key genomic and clinicopathologic findings, such as the association of gene fusions with more aggressive disease, and the finding of recurrent *DICER1* variants should help inform individualized treatment for patients and expand the understanding of the genetic mechanisms of thyroid tumors in children.

## Notes

Supplement. eMethods. eTable 1. Malignancy details eTable 2. Mutational annotation of thyroid nodules eTable 3. Clinicopathologic presentation and disease outcomes in pediatric patients with malignancies harboring a fusion or a mutation eTable 4. Comparison of the genomic classifier test to pre-operative clinical modeling in malignancy detection eTable 5. Concordance of FFPE and FNA samples eTable 6. Details of false negative samples eTable 7. Details of false positive samples eFigure 1. Oncoplot of 95 pediatric thyroid nodules subjected to comprehensive genomic testing eFigure 2. Gene fusions identified in malignant pediatric thyroid nodules eFigure 3. Survival of pediatric patients with fusion or mutation positive nodules eFigure 4. TSHR mutations are linked to NIS expression and pre-operative TSH levels eFigure 5. Spectrum of DICER1 mutations in pediatric thyroid nodules eFigure 6. STARD flow diagram eFigure 7. Genomic classification of pediatric thyroid nodules eFigure 8. Genomic probability of malignancy by pediatric thyroid nodule pathology eReferences.
