# Supplementary material for: Evaluation of the Molecular Landscape of Pediatric Thyroid Nodules and Use of a Multigene Genomic Classifier in Children
Source: JAMA Oncol. 2022 Jun 9;8(9):1323–7. doi: 10.1001/jamaoncol.2022.1655 (PMC9185516; doi:10.1001/jamaoncol.2022.1655)
Supplement: Supplement. — eMethods. eTable 1. Malignancy details eTable 2. Mutational annotation of thyroid nodules eTable 3. Clinicopathologic presentation and disease outcomes in pediatric patients with malignancies harboring a fusion or a mutation eTable 4. Comparison of the genomic classifier test to pre-operative clinical modeling in malignancy detection eTable 5. Concordance of FFPE and FNA samples eTable 6. Details of false negative samples eTable 7. Details of false positive samples eFigure 1. Oncoplot of 95 pediatric thyroid nodules subjected to comprehensive genomic testing eFigure 2. Gene fusions identified in malignant pediatric thyroid nodules eFigure 3. Survival of pediatric patients with fusion or mutation positive nodules eFigure 4. TSHR mutations are linked to NIS expression and pre-operative TSH levels eFigure 5. Spectrum of DICER1 mutations in pediatric thyroid nodules eFigure 6. STARD flow diagram eFigure 7. Genomic classification of pediatric thyroid nodules eFigure 8. Genomic probability of malignancy by pediatric thyroid nodule pathology eReferences. [file jamaoncol-e221655-s001.pdf]

Supplemental Online Content

Gallant JN, Chen SC, Ortega CA, et al. Evaluation of the molecular landscape of pediatric thyroid nodules and use of multigene genomic classifier in children. *JAMA Oncology*. Published online June 9, 2022. doi:10.1001/jamaoncol.2022.1655

eMethods.
eTable 1. Malignancy details
eTable 2. Mutational annotation of thyroid nodules
eTable 3. Clinicopathologic presentation and disease outcomes in pediatric patients with malignancies harboring a fusion or a mutation
eTable 4. Comparison of the genomic classifier test to pre-operative clinical modeling in malignancy detection
eTable 5. Concordance of FFPE and FNA samples
eTable 6. Details of false negative samples
eTable 7. Details of false positive samples
eFigure 1. Oncoplot of 95 pediatric thyroid nodules subjected to comprehensive genomic testing
eFigure 2. Gene fusions identified in malignant pediatric thyroid nodules
eFigure 3. Survival of pediatric patients with fusion or mutation positive nodules
eFigure 4. *TSHR* mutations are linked to NIS expression and pre-operative TSH levels
eFigure 5. Spectrum of *DICER1* mutations in pediatric thyroid nodules
eFigure 6. STARD flow diagram
eFigure 7. Genomic classification of pediatric thyroid nodules
eFigure 8. Genomic probability of malignancy by pediatric thyroid nodule pathology
eReferences.

This supplemental material has been provided by the authors to give readers additional information about their work.

© 2022 Gallant JN et al. *JAMA Oncology*.

eMethods.

Variable Details

Patient demographics, clinical histories, and outcomes were manually extracted from the electronic health record. Race and ethnicity, self-reported in our clinic intake forms, were gathered due to their potential confounding effect on outcomes[1]. Tumor pathology was classified according to the American Joint Committee on Cancer (AJCC) 8th edition[2]. Pre-operative thyroid nodule volumes were calculated based on ultrasound measurements and the ellipsoid formula ([length × width × depth] × [π / 6]). Body mass index z-scores were calculated using the R package zscorer as previously described.[3] American Thyroid Association (ATA) risk categories were determined using the latest pediatric guidelines and the definition of extensive disease provided in the adult guidelines (extensive involvement if > 5 lymph nodes or size of ≥ 3 cm in largest diameter).[4,5] Treatment outcomes and survival were defined per the ATA.[4,5]

Molecular Test

ThyroSeq v3 Genomic Classifier (GC) is a DNA/RNA next-generation sequencing (NGS) test. It uses targeted-amplification-based NGS technology to interrogate 12,135 single-nucleotide variants (SNVs) and insertions/deletions (indels) in 112 thyroid-cancer-related genes. The SNVs and indels are centered around hotspot mutations from the Catalogue of Somatic Mutations in Cancers[6]. The 112 thyroid-cancer-related gene list has been curated over several iterations of ThyroSeq and previously published[7–9]. The test also interrogates more than 120 gene fusion types, abnormal gene expression alterations (GEAs) of 19 genes, and copy number alterations (CNAs) in 10 genomic regions in fine needle aspiration (FNA) samples and in up to 27 genomic regions in (formalin-fixed paraffin-embedded [FFPE]) tissue samples[9]. ThyroSeq v3 has been extensively validated in several large multi-institutional studies for adult thyroid cancer[10,11]. ThyroSeq v3 was performed at the University of Pittsburgh Medical Center (UPMC) Molecular and Genomic Pathology laboratory per established protocols.[9] For FFPE (formalin-fixed paraffin-embedded) tissues (n = 132), six 4-5 μM unstained slides were submitted from Vanderbilt University Medical Center (VUMC). For fixed FNA cytology smear samples (n = 31), a variety of Diff-Quik (n = 32), Papanicolaou (n = 13), and Hematoxylin & Eosin (n = 14) stained slides were sent for testing. Testing of FNA cytology smear samples required 1 to 2 slides with at least 100 cells on a slide. All slides were digitally scanned for electronic archival preservation prior to sequencing.

Diagnostic Outcomes

The primary (diagnostic) outcome was the accuracy of the multigene GC (to predict the final surgical histopathologic diagnosis of benign versus malignant nodules) as measured by sensitivity, specificity, negative predictive value (NPV), positive predictive value (PPV), and under the receiver-operating characteristic (ROC) curve (AUC). In data analysis, noninvasive follicular thyroid neoplasm with papillary-like nuclear features (NIFTP) was grouped together with malignancies because it also represents a tumor type that requires surgery based on current practice guidelines.[5,12] For the final study set, only a single surgical (FFPE) sample per patient was analyzed to avoid duplicate statistical analysis of matched FNA samples. For secondary investigations, poor outcomes were defined as disease progression, recurrence, or persistence per ATA guidelines.[4,5] Poorly differentiated thyroid carcinoma (PDC) was also considered a poor outcome.

Statistical Analysis

Baseline descriptive statistics were summarized using medians (IQRs) and frequencies (percentages) and were compared across groups using Wilcoxon rank-sum (for continuous variables), Pearson's χ^2 , and Fisher's exact (for categorical variables) tests, respectively. Concordance of paired FFPE and FNA samples was tested via McNemar's and Cohen's kappa tests. Survival curves were estimated using the Kaplan-Meier method and compared between groups. Malignancy was modelled using logistic regression. Risk factors considered in the pre-operative clinical model include age, sex, ethnicity, BMI z-score, TSH, and nodule volume. Missing covariates were multiply imputed using multiple imputation by chained equation. Statistical significance was assessed at a 2-sided 5% level. The discrimination of each model was assessed using Harrell's C statistic and its 95% CI via bootstrapping. The C statistic is a rank-order statistic for predictions against true outcomes, with values ranging from 0.5 (no discrimination) to a theoretical maximum of 1.0. Sensitivity, specificity, PPV, NPV, prevalence, and AUC of the multigene GC are reported with 95% CIs. All statistical analyses were conducted with the R software package (version 4.1.1, R Foundation).

© 2022 Gallant JN et al. *JAMA Oncology*.

	All Malignancies (n = 50)
Histology, n (%)	
PTC variant	40 (80)
Classical	30 (75)
Diffuse sclerosing	4 (10)
Follicular	3 (8)
Tall cell	1 (3)
Solid	2 (5)
FTC	4 (8)
PDC	3 (6)
NIFTP	3 (6)
TNM[a], n (%)	
T1	16 (32)
T1a	8 (50)
T1b	8 (50)
T2	13 (26)
T3	15 (30)
T3a	8 (53)
T3b	7 (47)
T4	6 (12)
T4a	6 (100)
NX	10 (20)
N0	8 (16)
N1	32 (64)
N1a	12 (38)
N1b	20 (62)
MX	3 (6)
M0	43 (86)
M1	4 (8)
Longest tumor diameter, cm, median (IQR)	2.75 (1.5–4.1)
Adverse pathologic features present, n (%)	34 (68)
Vascular invasion	27 (54)
Extracapsular spread	22 (44)
Positive surgical margins	15 (30)
Initial treatments per patient, median (IQR)	2 (2–3)
Surgery	1 (1–2)
RAI	1 (0.2–1)
Poor patient outcome, n (%)	15 (30)
PDC	1 (7)
Persistent disease	5 (33)
Progression[b]	3 (20)
Recurrence[b]	6 (40)
DFS, months, median (IQR)	43 (20–60)
Additional treatments[b], n (%)	6 (66)
Surgery	5 (55)
RAI	2 (22)

eTable 1. Malignancy details

Abbreviations: PTC, papillary thyroid carcinoma; FTC, follicular thyroid carcinoma; PDC, poorly differentiated carcinoma; NIFTP, noninvasive follicular thyroid neoplasm with papillary-like nuclear features; TNM, TNM Classification of Malignant Tumors; IQR, interquartile range; RAI, radioactive iodine; DFS, disease free survival

[a] per the American Joint Committee on Cancer 8th edition

[b] includes 1 case of PDC

[b] in case of recurrence or progression; n out of 9 patients

© 2022 Gallant JN et al. *JAMA Oncology*.

Sample ID	Pathology	Hugo Symbol	Variant Classification	Nucleotide change	Protein change
20-10	PTC	RET	Fusion	—	CCDC6-RET (RET/PTC1)
20-11	PTC	RET	Fusion	—	CCDC6-RET (RET/PTC1)
20-12	PTC	BRAF	Mutation	c.1799T>A	p.V600E
20-14	FA	BRAF	Mutation	c.1397G>T	p.G466V
20-15	PTC	RET	Fusion	—	CCDC6-RET (RET/PTC1)
20-16	FA	NRAS	Mutation	c.181C>A	p.Q61K
20-17	FC	DICER1	Mutation	c.5126A>G	p.D1709G
20-2	PTC	RET	Fusion	—	NCOA4-RET (RET/PTC3)
20-20	FA	TSHR	Mutation	c.1897G>T	p.D633Y
20-21	PTC	RET	Fusion	—	CCDC6-RET (RET/PTC1)
20-22	PTC	RET	Fusion	—	NCOA4-RET (RET/PTC3)
20-24	PTC	BRAF	Mutation	c.1799T>A	p.V600E
20-244	FA	PTEN	Mutation	c.253+1G>A	p.splice
20-249	PTC	RET	Fusion	—	GOLGA5-RET
20-25	PDC	DICER1	Mutation	c.5125G>A	p.D1709N
20-255	PTC	NTRK3	Fusion	—	ETV6-NTRK3
20-257	PTC	RET	Fusion	—	NCOA4-RET (RET/PTC3)
20-259	FA	TSHR	Mutation	c.1358T>C	p.M453T
20-260	FA	TSHR	Mutation	c.1535T>G	p.L512R
20-262	MNG	TSHR	Mutation	c.1535T>G	p.L512R
20-264	MNG	TSHR	Mutation	c.1358T>C	p.M453T
20-269	FA	TSHR	Mutation	c.1918A>G	p.I640V
20-270	FA	TSHR	Mutation	c.1703T>C	p.I568T
20-272	MNG	DICER1	Mutation	c.5113G>A	p.E1705K
20-273	MNG	RET	Mutation	c.1858T>C	p.C620R
20-274	MNG	TSHR	Mutation	c.1514G>A	p.S505N
20-276	MNG	TSHR	Mutation	c.1456A>T	p.I486F
20-277	MNG	TSHR	Mutation	c.1358T>C	p.M453T
20-278	PDC	DICER1	Mutation	c.5437G>A	p.E1813K
20-28	PTC	RET	Fusion	—	CCDC6-RET (RET/PTC1)
20-280	MNG	GNAS	Mutation	c.681G>C	p.Q227H
20-284	MNG	TSHR	Mutation	c.1456A>T	p.I486F
20-285	MNG	TSHR	Mutation	c.1897G>T	p.D633Y
20-287	PTC	RET	Fusion	—	CCDC6-RET (RET/PTC1)
20-289	MNG	DICER1	Mutation	c.5438A>G	p.E1813G
20-29	FA	DICER1	Mutation	c.5439G>T	p.E1813D
20-290	PTC	RET	Fusion	—	CCDC6-RET (RET/PTC1)
20-292	MNG	TSHR	Mutation	c.1456A>T	p.I486F
20-294	PTC	RET	Fusion	—	NCOA4-RET (RET/PTC3)
20-295	PTC	RET	Fusion	—	CCDC6-RET (RET/PTC1)
20-297	MNG	TSHR	Mutation	c.1456A>T	p.I486F
20-298	MNG	TSHR	Mutation	c.1535T>G	p.L512R
20-299	PTC	NTRK1	Fusion	—	TPM3-NTRK1
20-3	PTC	ALK	Fusion	—	STRN-ALK
20-30	PTC	NRAS	Mutation	c.37G>C	p.G13R
20-300	PDC	PPARG	Fusion	—	PAX8-PPARG
20-301	PTC	RET	Fusion	—	CCDC6-RET (RET/PTC1)
20-302	FC	TSHR	Mutation	c.1358T>C	p.M453T
20-303	PTC	RET	Fusion	—	CCDC6-RET (RET/PTC1)
20-304	PTC	BRAF	Mutation	c.1799T>A	p.V600E
20-305	PTC	RET	Fusion	—	CCDC6-RET (RET/PTC1)
20-32	PTC	RET	Fusion	—	CCDC6-RET (RET/PTC1)
20-33	FA	TSHR	Mutation	c.1893C>G	p.F631L
20-34	FA	DICER1	Mutation	c.5113G>A	p.E1705K
20-35	FA	PTEN	Mutation	c.1003C>T	p.R335*
20-35	FA	PTEN	Mutation	c.752G>T	p.G251V
20-36	FA	TSHR	Mutation	c.1535T>G	p.L512R

© 2022 Gallant JN et al. *JAMA Oncology*.

Sample ID	Pathology	Hugo Symbol	Variant Classification	Nucleotide change	Protein change
20-37	PTC	BRAF	Mutation	c.1799T>A	p.V600E
20-38	FC	GNAS	Mutation	c.602G>A	p.R201H
20-39	NIFTP	HRAS	Mutation	c.182A>G	p.Q61R
20-4	PTC	BRAF	Fusion		ZC3HAV1-BRAF
20-40	PTC	GNAS	Mutation	c.681G>C	p.Q227H
20-41	NIFTP	NRAS	Mutation	c.181C>A	p.Q61K
20-44	PTC	RET	Fusion	—	NCOA4-RET (RET/PTC3)
20-45	PTC	RET	Fusion	—	NCOA4-RET (RET/PTC3)
20-46	FA	TSHR	Mutation	c.1358T>C	p.M453T
20-47	PTC	BRAF	Mutation	c.1799T>A	p.V600E
20-48	PTC	RET	Fusion	—	CCDC6-RET (RET/PTC1)
20-49	PTC	BRAF	Mutation	c.1799T>A	p.V600E
20-5	PTC	RET	Fusion	—	NCOA4-RET (RET/PTC3)
20-50	PTC	BRAF	Mutation	c.1799T>A	p.V600E
20-51	FA	PPARG	Fusion	—	PAX8-PPARG
20-52	FA	NRAS	Mutation	c.181C>A	p.Q61K
20-53	FA	DICER1	Mutation	c.5437G>C	p.E1813Q
20-54	FC	DICER1	Mutation	c.5437G>C	p.E1813Q
20-55	FA	TSHR	Mutation	c.1897G>C	p.D633H
20-56	FA	DICER1	Mutation	c.5126A>G	p.D1709G
20-7	PTC	NTRK3	Fusion	—	ETV6-NTRK3
20-8	PTC	RET	Fusion	—	CCDC6-RET (RET/PTC1)
20-9	PTC	RET	Fusion	—	NCOA4-RET (RET/PTC3)
21-33	NIFTP	KRAS	Mutation	c.182A>G	p.Q61R

eTable 2. Mutational annotation of thyroid nodules
Mutation annotation format style overview of pediatric thyroid nodule mutations and fusions. Abbreviations: MNG, multinodular goiter; FA, follicular adenoma; NIFTP, noninvasive follicular thyroid neoplasm with papillary-like nuclear features; FTC, follicular thyroid carcinoma; PDC, poorly differentiated carcinoma; PTC, papillary thyroid carcinoma (and variants).

© 2022 Gallant JN et al. *JAMA Oncology*.

	Fusion positive (n = 29)	Mutation positive (n = 18)	P-value[a]
Age at surgery, years, median (IQR)	14.4 (11.6–16.3)	16.8 (16.1–17.5)	<0.01[a]
Sex, n (%)			0.47[c]
Female	24 (83)	13 (72)	
Male	5 (17)	5 (28)	
Race, n (%)			0.99[c]
White	25 (86)	16 (89)	
Non-white	4 (14)	2 (11)	
Histology, n (%)			<0.01[c]
PTC	28 (97)	9 (50)	
FTC	0 (0)	4 (22)	
PDC	1 (3)	2 (11)	
NIFTP	0 (0)	3 (16)	
TNM, n (%)			
T1/T2	11 (38)	15 (83)	<0.01[b]
T3/T4	18 (62)	3 (17)	
N0/NX	4 (14)	13 (72)	<0.01[c]
N1+	25 (86)	5 (28)	
M0/MX	26 (90)	17 (94)	0.40[c]
M1	3 (10)	1 (6)	
Adverse pathologic features present, n (%)	21 (72)	12 (67)	0.42[b]
Vascular invasion	20 (69)	6 (33)	0.02[b]
Extracapsular spread	14 (48)	7 (39)	0.53[b]
Positive surgical margins	12 (41)	3 (17)	0.08[b]
Initial treatments per patient, median (range)	2 (1–5)	2 (1–4)	<0.01[a]
Surgery	1 (1–3)	1 (1–3)	0.64[a]
RAI	1 (0–3)	0 (0–1)	<0.01[a]
Poor patient outcome, n (%)	10 (34)	4 (22)	0.37[b]
Persistent disease	5 (50)	0 (0)	0.14[c]
Progression	2 (20)	1 (25)	0.99[c]
Recurrence	3 (30	3 (75)	0.67[c]

eTable 3. Clinicopathologic presentation and disease outcomes in pediatric patients with malignancies harboring a fusion or a mutation

Abbreviations: IQR, interquartile range; PTC, papillary thyroid carcinoma; FTC, follicular thyroid carcinoma; PDC, poorly differentiated carcinoma; NIFTP, noninvasive follicular thyroid neoplasm with papillary-like nuclear features; TNM, TNM Classification of Malignant Tumors per the American Joint Committee on Cancer 8th edition; RAI, radioactive iodine.

[a] Wilcoxon rank sum test

[b] Pearson's Chi-square test

[c] Fisher's exact test

© 2022 Gallant JN et al. *JAMA Oncology*.

	Pre-operative clinical model[a]		Genomic classifier test	
	C Statistic	95% CI	C Statistic	95% CI
Original	0.75	NA	0.87	NA
Training	0.77	0.68–0.86	0.87	0.81–0.93
Test	0.65	0.50–0.81	0.87	0.78–0.97

eTable 4. Comparison of the genomic classifier test to pre-operative clinical modeling in malignancy detection
Comparison of the C Statistic (AUC) with bootstrapped 95% confidence intervals of the best possible pre-operative clinical model as compared to the genomic classifier test. Test C statistics were calculated and corrected for possible optimism using internal bootstrap resampling with 500 replicates. Abbreviations: CI; confidence interval
[a] incorporates pre-clinical factors in Table 1, including age, sex, ethnicity, BMI z-score, TSH, and nodule volume

© 2022 Gallant JN et al. *JAMA Oncology*.

Positive GC

		FFPE samples		Proportion		Test	
		Positive	Negative	FNA	FFPE	McNemar stat = 1	Cohen's kappa = 0.862
FNA samples	Positive	18	0	0.783	0.826	p = 0.317	p < 0.001
	Negative	1	4				

Positive mutation

		FFPE samples		Proportion		Test	
		Positive	Negative	FNA	FFPE	McNemar stat = 1	Cohen's kappa = 0.911
FNA samples	Positive	13	0	0.565	0.609	p = 0.317	p < 0.001
	Negative	1	9				

Positive CNA

		FFPE samples		Proportion		Test	
		Positive	Negative	FNA	FFPE	McNemar stat = 1	Cohen's kappa = 0.646
FNA samples	Positive	1	0	0.043	0.087	p = 0.317	p < 0.001
	Negative	1	21				

Positive fusion

		FFPE samples		Proportion		Test	
		Positive	Negative	FNA	FFPE	McNemar stat = NA	Cohen's kappa = 1.000
FNA samples	Positive	8	0	0.364	0.364	p = NA	p < 0.001
	Negative	0	14				

Positive GEA

		FFPE samples		Proportion		Test	
		Positive	Negative	FNA	FFPE	McNemar stat = 2	Cohen's kappa = 0.820
FNA samples	Positive	10	0	0.455	0.545	p = 0.157	p < 0.001
	Negative	2	10				

eTable 5. Concordance of FFPE and FNA samples
Abbreviations: GC, genomic classifier; CNA, copy number alterations; GEA, gene enrichment analysis.

© 2022 Gallant JN et al. *JAMA Oncology*.

Sample ID	Diagnosis	TNM	ATA	Mutation	CNA	Fusion	GEA	NIS
20-23	PTC	T1bN1aM0	Low	Negative	Negative	Negative	Negative	0%
20-302	FTC	T2N0M0	Low	TSHR G466V	Negative	Negative	Negative	10%

eTable 6. Details of false negative samples
Abbreviations: PTC, papillary thyroid carcinoma; FTC, follicular thyroid carcinoma; TNM, TNM Classification of Malignant Tumors per the American Joint Committee on Cancer 8th edition; ATA; American Thyroid Association pediatric thyroid cancer risk level; CNA, copy number alteration; GEA, gene enrichment analysis; NIS, sodium-iodide symporter expression percentage.

© 2022 Gallant JN et al. *JAMA Oncology*.

Sample ID	Diagnosis	Mutation	CNA	Fusion	GEA	NIS
20-16	FA	NRAS Q61K	Negative	Negative	Negative	0%
20-272	MNG	DICER1 E1705K	Negative	Negative	Negative	0%
20-283	MNG	Negative	High	Negative	Negative	5%
20-289	MNG	DICER1 E1813G	Negative	Negative	Negative	0%
20-29	FA	DICER1 E1813D	Negative	Negative	Negative	0%
20-34	FA	DICER1 E1705K	Negative	Negative	Negative	11%
20-51	FA	Negative	Negative	*PAX8–PPARG*	Negative	0%
20-52	FA	NRAS Q61K	Negative	Negative	Positive	0%
20-53	FA	DICER1 E1813Q	Negative	Negative	Negative	0%
20-56	FA	DICER1 D1709G	Negative	Negative	Negative	3%

eTable 7. Details of false positive samples
Abbreviations: MNG, multinodular goiter; FA, follicular adenoma; CNA, copy number alteration; GEA, gene enrichment analysis; NIS, sodium-iodide symporter expression percentage.

© 2022 Gallant JN et al. *JAMA Oncology*.

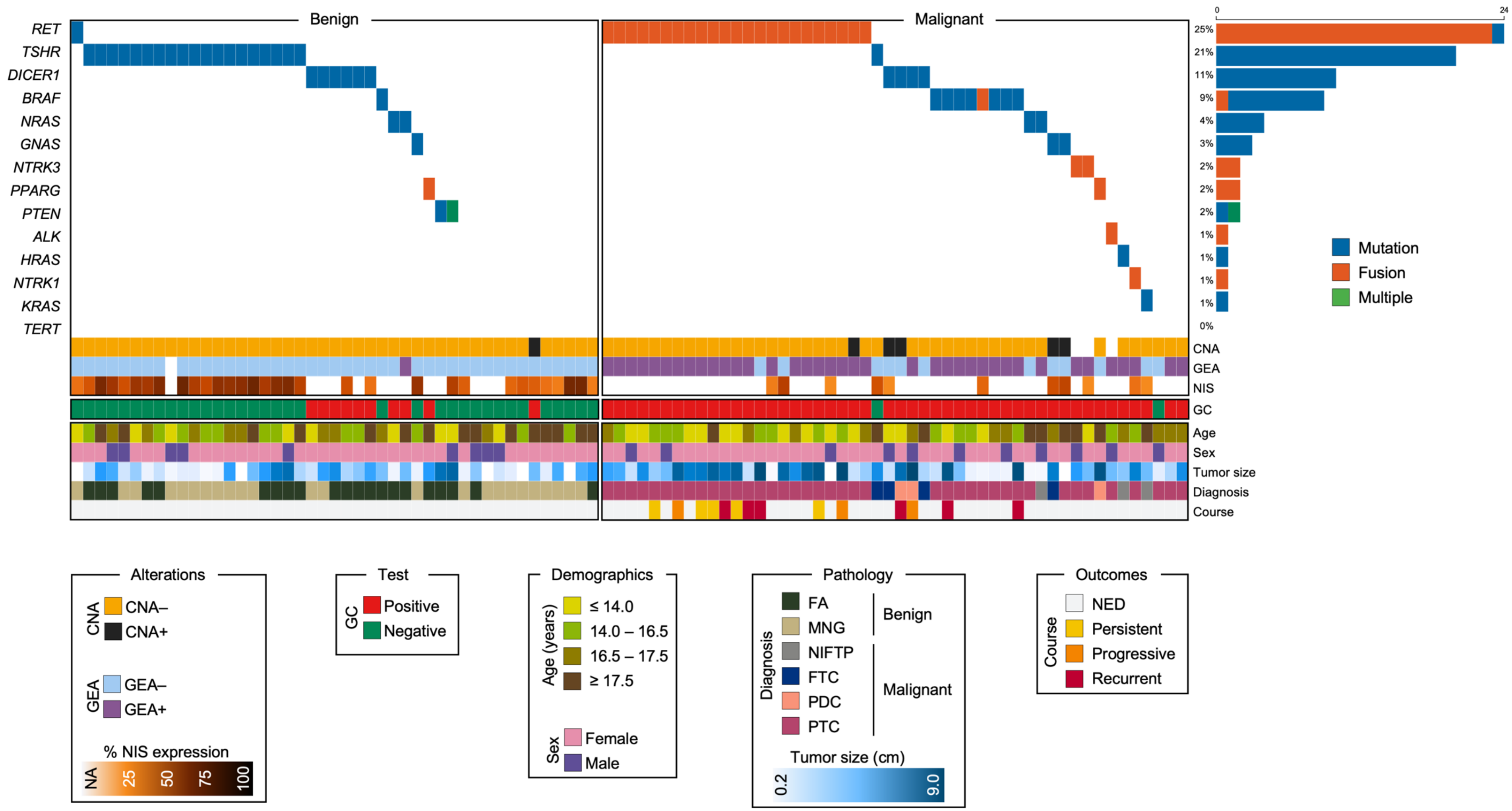

eFigure 1. Oncoplot of 95 pediatric thyroid nodules subjected to comprehensive genomic testing
Recurrent mutations in genes (left) ranked by frequency (right) and separated by pathology (bottom). Additional alteration, demographic, pathology, and outcome data are shown in corresponding tile plots. Abbreviations: CNA, copy number alterations; GEA, gene expression alterations; NIS, sodium iodide symporter; GC, genomic classifier result; MNG, multinodular goiter; FA, follicular adenoma; NIFTP, noninvasive follicular thyroid neoplasm with papillary-like nuclear features; FTC, follicular thyroid carcinoma; PDC, poorly differentiated carcinoma; PTC, papillary thyroid carcinoma (and variants); NED, no evidence of disease. The benign *RET* mutation was identified in a prophylactic thyroidectomy sample.

© 2022 Gallant JN et al. *JAMA Oncology*.

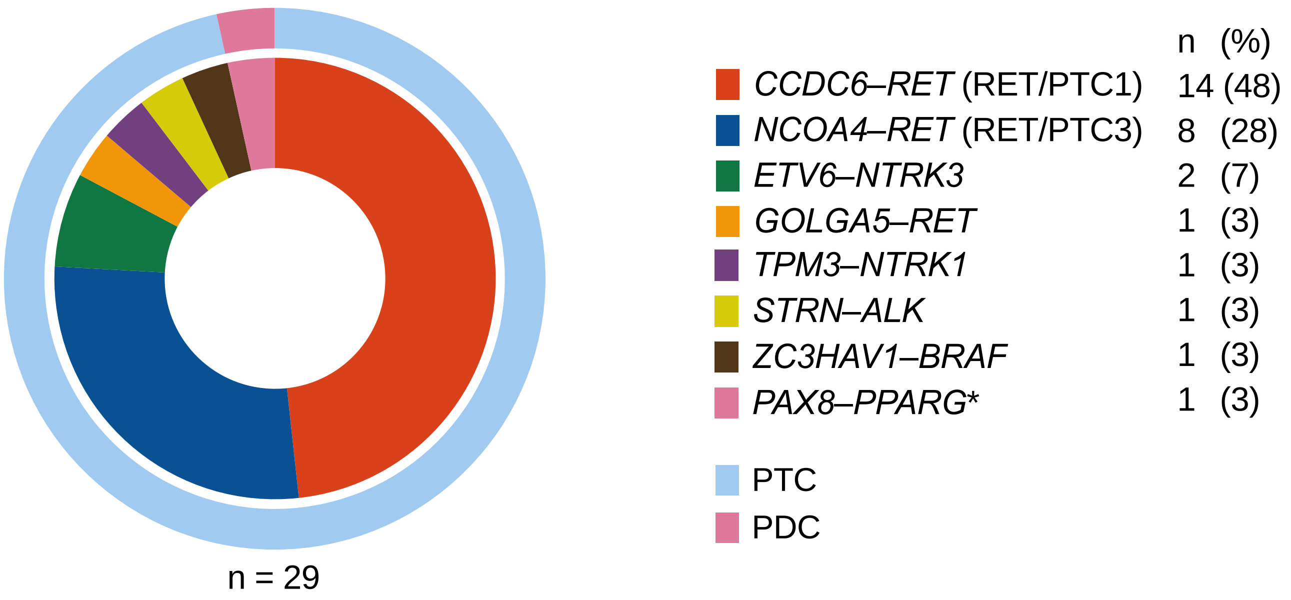

eFigure 2. Gene fusions identified in malignant pediatric thyroid nodules
Pie chart demonstrating the breakdown of gene fusions identified in pediatric malignant thyroid nodules. The inner ring breaks down the different gene fusions, and the outer ring highlights the nodule pathology. Abbreviations: PTC, papillary thyroid cancer (and variants); PDC, poorly differentiated carcinoma. The *PAX8-PPARG* (asterisk) fusion was also identified in a benign nodule (follicular adenoma).

© 2022 Gallant JN et al. *JAMA Oncology*.

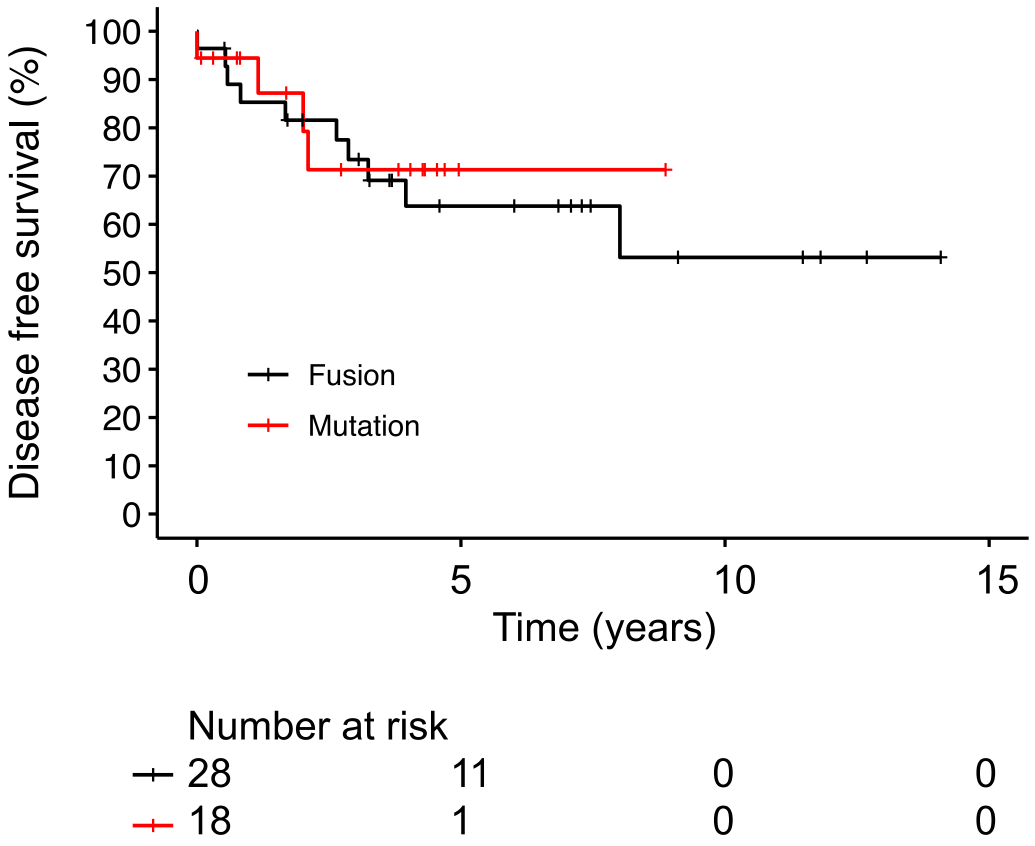

eFigure 3. Survival of pediatric patients with fusion or mutation positive nodules
Kaplan-Meier analysis showing no difference in disease-free survival probability between patients whose malignant nodules harbored a fusion (black) or mutation (red).

© 2022 Gallant JN et al. *JAMA Oncology*.

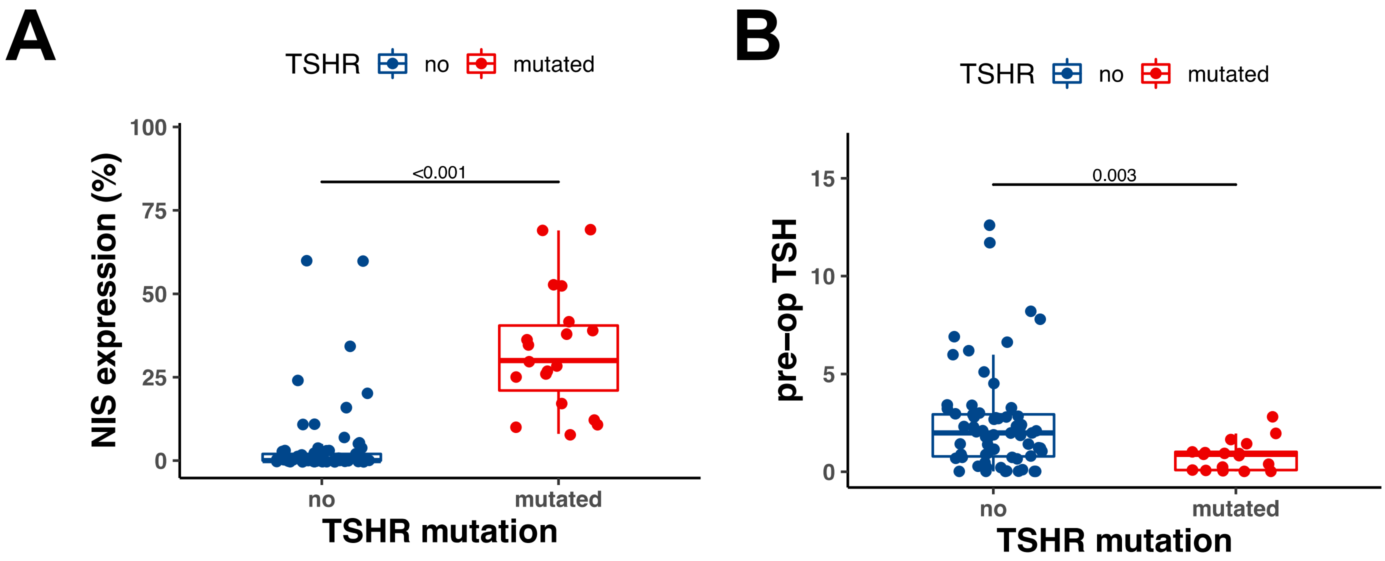

eFigure 4. *TSHR* mutations are linked to NIS expression and pre-operative TSH levels
Box and whisker plots comparing TSHR (mutations to NIS expression (**A**) and pre-operative TSH levels (**B**) in benign nodules. Abbreviations: NIS, sodium-iodide symporter; TSH, thyroid stimulating hormone.

© 2022 Gallant JN et al. *JAMA Oncology*.

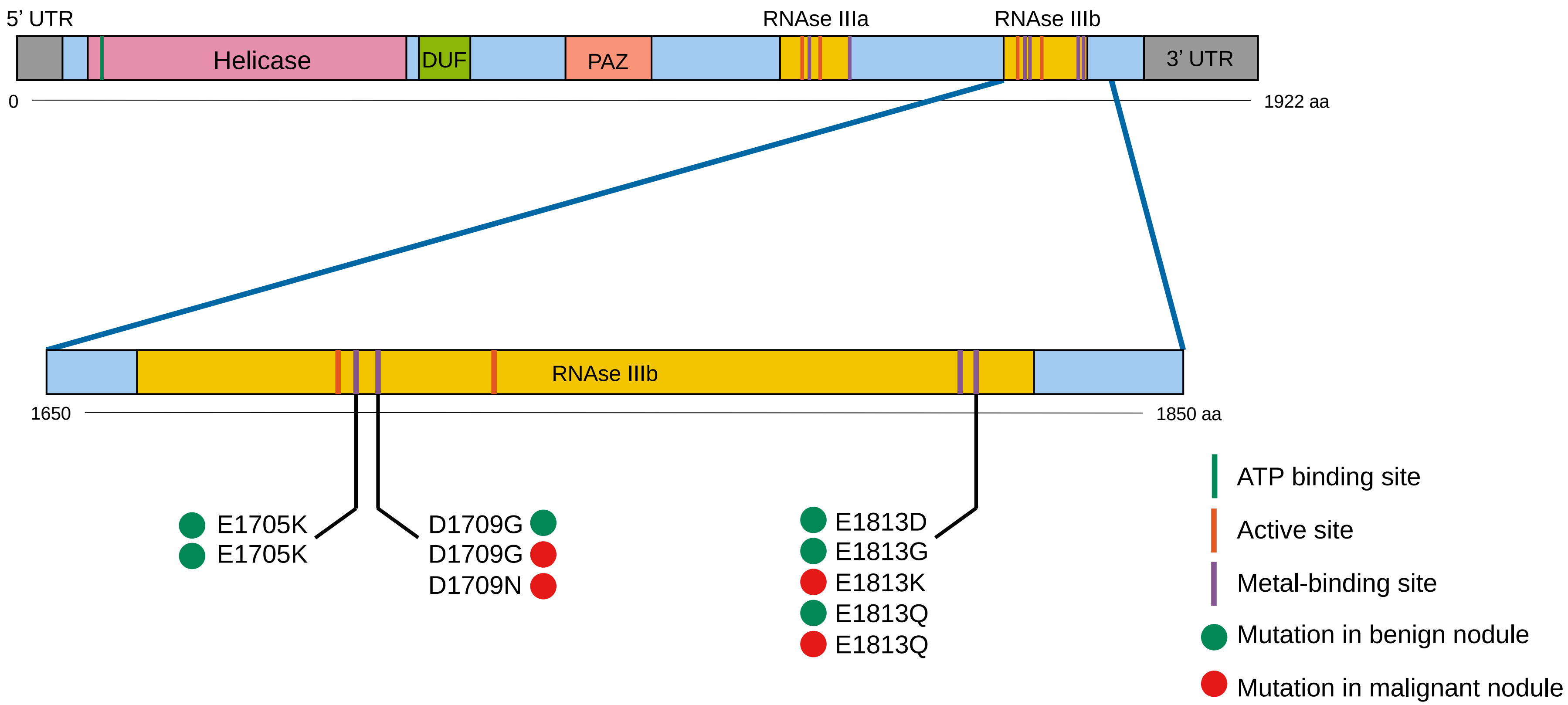

eFigure 5. Spectrum of *DICER1* mutations in pediatric thyroid nodules
Schematic of the DICER1 protein with the two catalytic RNase III domains shown in light yellow. Active sites are indicated by an orange line, and metal-binding sites are indicated by a purple line. Abbreviations: UTR, untranslated region; DUF, domain of unknown function; PAZ, Piwi Argonaut and Zwille domain; aa, amino acid. Green mutations were in benign nodules, while red mutations were in malignant nodules.

© 2022 Gallant JN et al. *JAMA Oncology*.

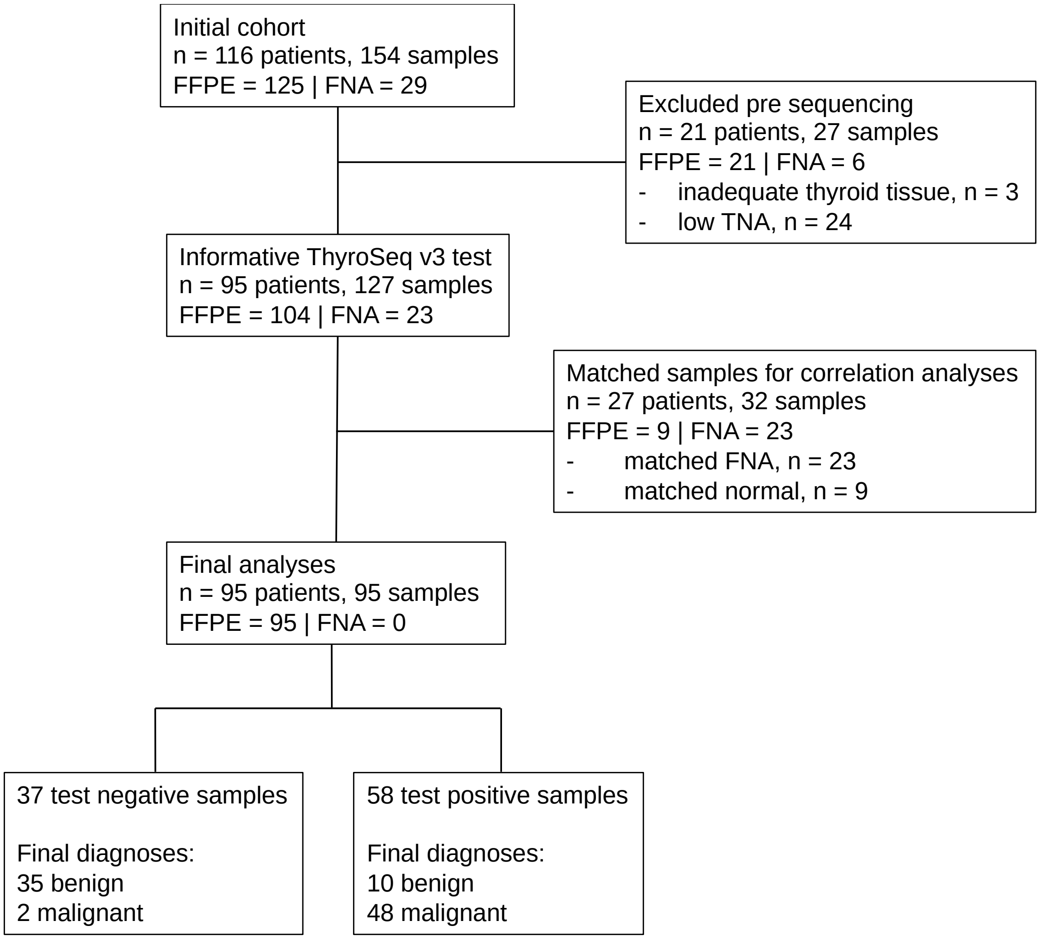

eFigure 6. STARD flow diagram

Standards for Reporting Diagnostic accuracy studies (STARD) diagram outlining recruitment/exclusion of patients/samples in the study. One hundred fifty-four samples from 116 patients were submitted. Twenty-seven (18%) samples (22 FFPE, 4 FNA) failed a pre-sequencing step owing to poor total nucleic acid quality. These failed samples were some of the oldest tested (mean [standard deviation, SD] 11.5 [1.5] years old) and significantly older than those that passed (mean [SD] 7.0 [0.9] years old; $p < 0.01$). Three samples (1 FFPE, 2 FNA) were excluded as inadequate thyroid lesion tissue was found on re-review of specimens. Twenty-three matching pairs of FNA were included in secondary analyses (for concordance testing, see **eTable5**) along with 9 matched normal tissues, the latter for testing of *DICER1* somatic mutational status. Ninety-five samples (one FFPE sample per patient) were included in primary test characteristic analyses. Abbreviations: FFPE, formalin-fixed paraffin-embedded tissue; FNA, fine needle aspiration sample; TNA, total nucleic acids.

© 2022 Gallant JN et al. *JAMA Oncology*.

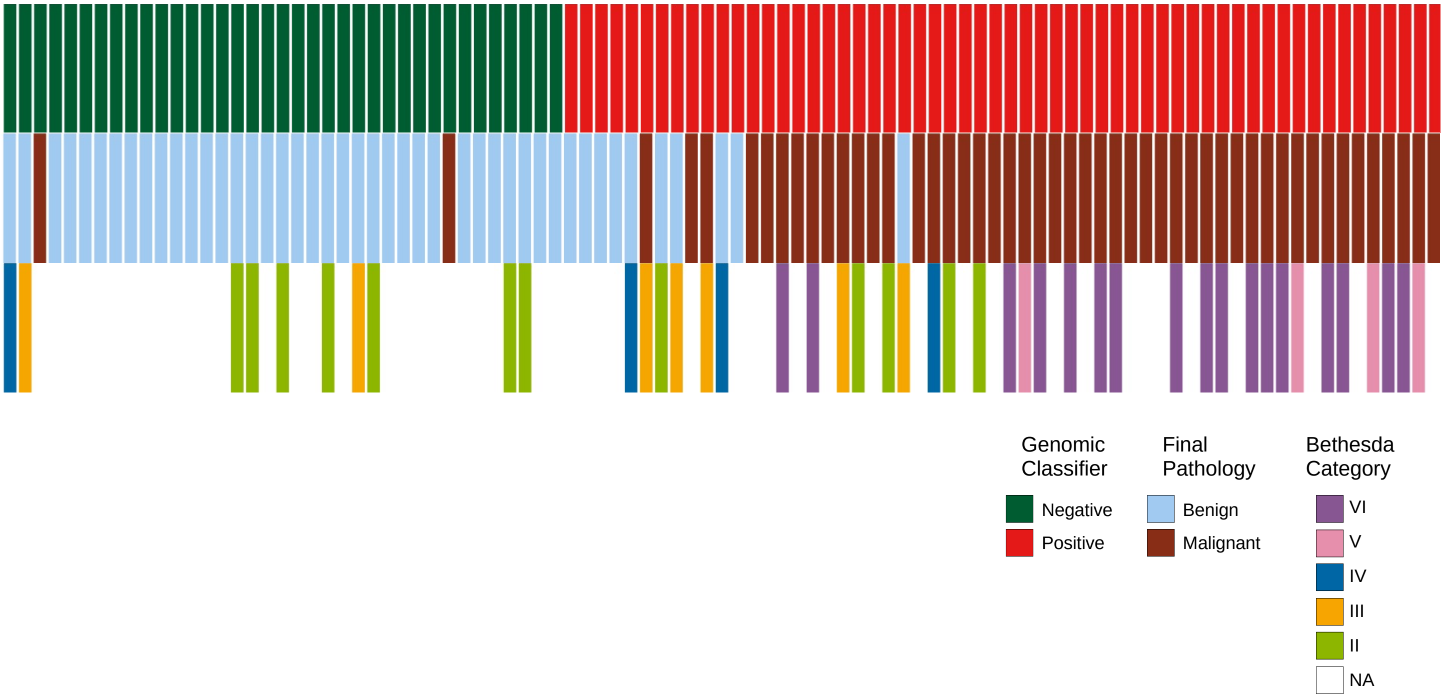

eFigure 7. Genomic classification of pediatric thyroid nodules
Tile plot comparing results of the genomic classifier, final pathology, and pre-operative FNAs per The Bethesda System for Reporting Thyroid Cytopathology: (NA) not performed; (II) benign; (III) atypia of undetermined significance (AUS) or follicular lesion of undetermined significance (FLUS); (IV) follicular neoplasm or suspicious for a follicular neoplasm; (V) suspicious for malignancy; and (VI) malignant.

© 2022 Gallant JN et al. *JAMA Oncology*.

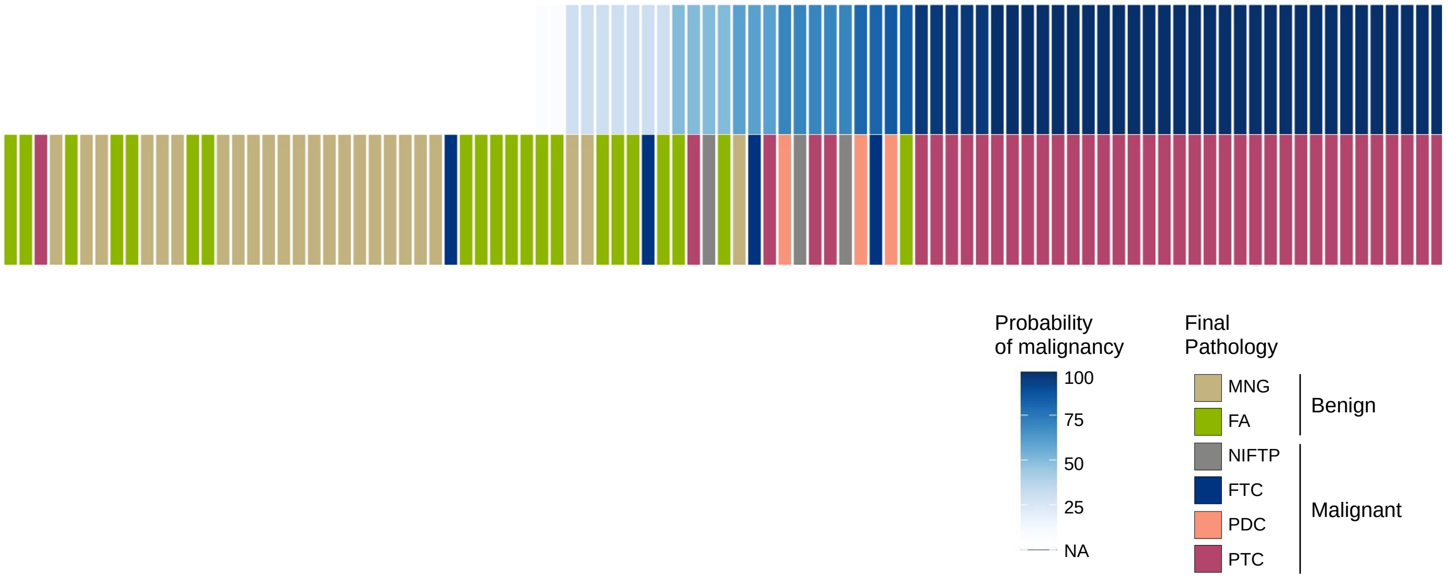

eFigure 8. Genomic probability of malignancy by pediatric thyroid nodule pathology
Tile plot comparing results of the genomic classifier's risk of malignancy and final surgical pathology. Abbreviations: MNG, multinodular goiter; FA, follicular adenoma; NIFTP, noninvasive follicular thyroid neoplasm with papillary-like nuclear features; FTC, follicular thyroid carcinoma, PDC, poorly differentiated carcinoma; PTC, papillary thyroid carcinoma

© 2022 Gallant JN et al. *JAMA Oncology*.

eReferences.

1. Gruszczynski NR, Low CM, Choby G, Meister KD, Smith BH, Balakrishnan K. Effects of Social Determinants of Health Care on Pediatric Thyroid Cancer Outcomes in the United States. *Otolaryngology Head Neck Surg*. Published online 2021:019459982110329. doi:10.1177/01945998211032901

2. Amin MB, Edge SB, Greene FL, et al., eds. *AJCC Cancer Staging Manual*. 8th ed. Springer International Publishing; 2017.

3. Ortega CA, Gallant JN, Chen SC, et al. Evaluation of Thyroid Nodule Malignant Neoplasms and Obesity Among Children and Young Adults. *Jama Netw Open*. 2021;4(7):e2116369. doi:10.1001/jamanetworkopen.2021.16369

4. Francis GL, Waguespack SG, Bauer AJ, et al. Management Guidelines for Children with Thyroid Nodules and Differentiated Thyroid Cancer. *Thyroid*. 2015;25(7):716-759. doi:10.1089/thy.2014.0460

5. Haugen BR, Alexander EK, Bible KC, et al. 2015 American Thyroid Association Management Guidelines for Adult Patients with Thyroid Nodules and Differentiated Thyroid Cancer: The American Thyroid Association Guidelines Task Force on Thyroid Nodules and Differentiated Thyroid Cancer. *Thyroid*. 2016;26(1):1-133. doi:10.1089/thy.2015.0020

6. Tate JG, Bamford S, Jubb HC, et al. COSMIC: the Catalogue Of Somatic Mutations In Cancer. *Nucleic Acids Res*. 2018;47(Database issue):gky1015-. doi:10.1093/nar/gky1015

7. Nikiforova MN, Wald AI, Roy S, Durso MB, Nikiforov YE. Targeted Next-Generation Sequencing Panel (ThyroSeq) for Detection of Mutations in Thyroid Cancer. *J Clin Endocrinol Metabolism*. 2013;98(11):E1852-E1860. doi:10.1210/jc.2013-2292

8. Nikiforov YE, Carty SE, Chiosea SI, et al. Highly accurate diagnosis of cancer in thyroid nodules with follicular neoplasm/suspicious for a follicular neoplasm cytology by ThyroSeq v2 next-generation sequencing assay. *Cancer*. 2014;120(23):3627-3634. doi:10.1002/cncr.29038

9. Nikiforova MN, Mercurio S, Wald AI, et al. Analytical performance of the ThyroSeq v3 genomic classifier for cancer diagnosis in thyroid nodules. *Cancer*. 2018;124(8):1682-1690. doi:10.1002/cncr.31245

10. Steward DL, Carty SE, Sippel RS, et al. Performance of a Multigene Genomic Classifier in Thyroid Nodules With Indeterminate Cytology. *Jama Oncol*. 2019;5(2):204-212. doi:10.1001/jamaoncol.2018.4616

11. Livhits MJ, Zhu CY, Kuo EJ, et al. Effectiveness of Molecular Testing Techniques for Diagnosis of Indeterminate Thyroid Nodules. *Jama Oncol*. 2021;7(1). doi:10.1001/jamaoncol.2020.5935

12. Nikiforov YE, Seethala RR, Tallini G, et al. Nomenclature Revision for Encapsulated Follicular Variant of Papillary Thyroid Carcinoma: A Paradigm Shift to Reduce Overtreatment of Indolent Tumors. *Jama Oncol*. 2016;2(8):1023. doi:10.1001/jamaoncol.2016.0386

© 2022 Gallant JN et al. *JAMA Oncology*.
